# Epigenetic Clocks: Beyond Biological Age, Using the Past to Predict the Present and Future

**DOI:** 10.14336/AD.2024.1495

**Published:** 2025-12-13

**Authors:** Runyu Liang, Qiang Tang, Jia Chen, Luwen Zhu

**Affiliations:** ^1^Heilongjiang University of Chinese Medicine, Harbin, China.; ^2^Second Affiliated Hospital of Heilongjiang University of Chinese Medicine, Harbin, China.

**Keywords:** Epigenesis, Genetic, DNA Methylation Aging, Biological Clocks, Biomarkers

## Abstract

Predicting health trajectories and accurately measuring aging processes across the human lifespan remain profound scientific challenges. Assessing the effectiveness and impact of interventions targeting aging is even more elusive, largely due to the intricate, multidimensional nature of aging—a process that defies simple quantification. Traditional biomarkers offer only partial perspectives, capturing limited aspects of the aging landscape. Yet, over the past decade, groundbreaking advancements have emerged. Epigenetic clocks, derived from DNA methylation patterns, have established themselves as powerful aging biomarkers, capable of estimating biological age and assessing aging rates across diverse tissues with remarkable precision. These clocks provide predictive insights into mortality and age-related disease risks, effectively distinguishing biological age from chronological age and illuminating enduring questions in gerontology. Despite significant progress in epigenetic clock development, substantial challenges remain, underscoring the need for continued investigation to fully unlock their potential in the science of aging.

## 1. Introduction

The study of aging extends beyond the pursuit of medical advancements; it represents a fundamental inquiry driven by our innate curiosity about life itself. Epigenetic has emerged as a crucial factor in modulating the aging process, providing insights beyond traditional genetics into how environmental factors shape the aging trajectory [1]. Over the past decade, major breakthroughs in epigenetic research have reframed our understanding of aging, positioning DNA—the core blueprint of life—as a repository of vital information capable of signaling future physiological states. DNA methylation, a key mechanism in epigenetic regulation, undergoes significant shifts with age, establishing it as a reliable indicator of biological aging [2, 3]. The potential to reverse these epigenetic alterations offers promising avenues for decelerating aging and possibly extending lifespan [4].

Epigenetic modifications encompass chemical changes to DNA or chromatin that influence gene expression and phenotype without altering the DNA sequence itself. These changes can be long-lasting and may even span generations [5]. Epigenetic clocks, which measure predictable changes in DNA methylation across the lifespan, have become invaluable tools for assessing biological aging. DNA methylation levels shift progressively in specific genomic regions, disrupting biological states in a predictable manner that exhibits clock-like behavior. These age-related methylation sites make up approximately 28% of the human genome [6, 7]. As “historical data” embedded within DNA, epigenetic clocks have demonstrated substantial predictive capabilities, offering unique insights into age-related health risks and enabling a clearer distinction between biological and chronological age.

Comparative studies have examined various potential age estimators, including telomere length, transcriptome profiles, proteomics, metabolomics, and composite biomarkers, consistently identifying epigenetic clocks as the most promising tools for biological age estimation [8, 9]. The advent of these clocks has provided researchers with objective metrics for evaluating the effectiveness of anti-aging interventions [10, 11].

**Figure 1.**
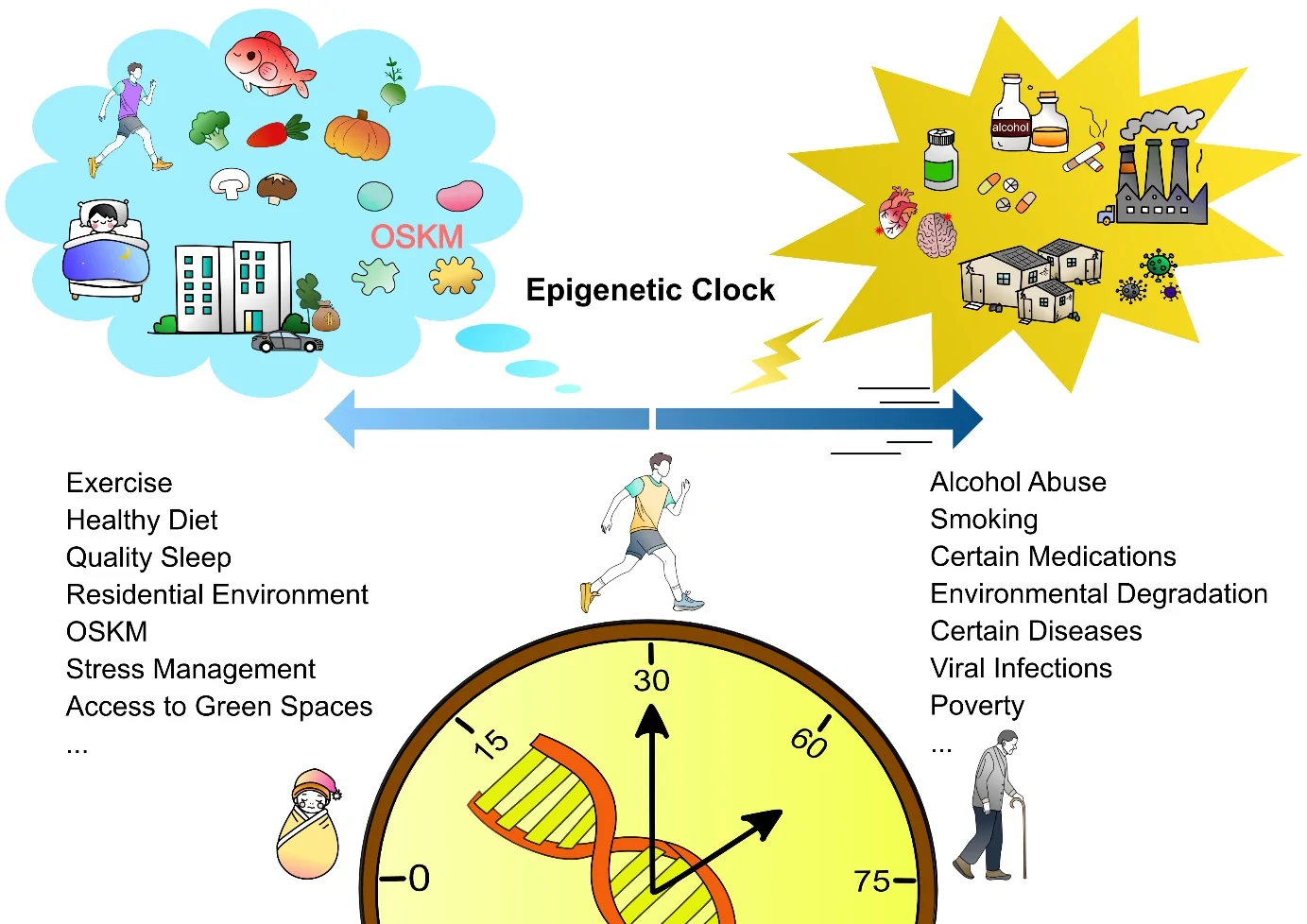
**Molecular changes associated with aging often drive observable changes at the macroscopic level, and epigenetic clocks can reflect these shifts**. Various factors may accelerate or decelerate the rate measured by epigenetic clocks, offering insights into the dynamic progression of aging. The elements and text on the left with a light blue background represent epigenetic clock deceleration events, while those on the right with a yellow background represent epigenetic clock acceleration events.

Despite considerable progress in the development of epigenetic clocks, substantial challenges remain. There is ongoing demand for more robust, precise, and context-specific models, particularly those attuned to age-related diseases and underlying drivers of aging. This review provides a comprehensive analysis of the development and applications of various epigenetic clocks, examining their strengths, limitations, and practical applications. By examining the strengths and limitations of these models, we aim to identify essential pathways for future research, driving novel approaches for early detection of age-related diseases and advancing tailored interventions in aging science (Fig. 1.)

## 2. Current Status of Epigenetic Clocks

Unlike chronological age, epigenetic clocks are biological tools based on DNA methylation patterns that estimate an individual’s biological age, offering deeper insights into the aging process [12]. In real-world settings, individuals of the same chronological age can show marked differences in epigenetic profiles. A younger-than-expected epigenetic age suggests slower aging, while an older-than-expected epigenetic age may indicate accelerated aging influenced by factors such as lifestyle, environment, and disease [13-17].

The development of epigenetic clocks has relied largely on large-scale DNA methylation datasets that reveal dynamic changes with age, especially at certain CpG sites. By identifying age-related CpG sites through regression and machine learning algorithms, researchers have constructed models that serve as accurate markers of biological age[18]. Epigenetic clocks are broadly categorized into two generations. The first generation, often referred to as “epigenetic age estimators,” focuses on estimating biological age, while the second generation, known as “phenotypic age,” clocks, incorporates additional risk factors to enhance predictions of health status, physiological changes, and aging rate.

However, the predictive accuracy of epigenetic clocks is affected by factors such as genetic background, lifestyle, environmental exposures, and technical variability [19]. Many existing clocks also have limitations in forecasting healthspan, assessing disease risk, and predicting cellular senescence [20-22]. Ongoing research is focused on optimizing predictive accuracy and improving the generalizability of these clocks across different populations and tissues [23]. Emerging technologies, such as single-cell methylation sequencing and multi-omics integration, are opening new possibilities for creating more precise and comprehensive epigenetic clocks [24-26].

**Figure 2.**
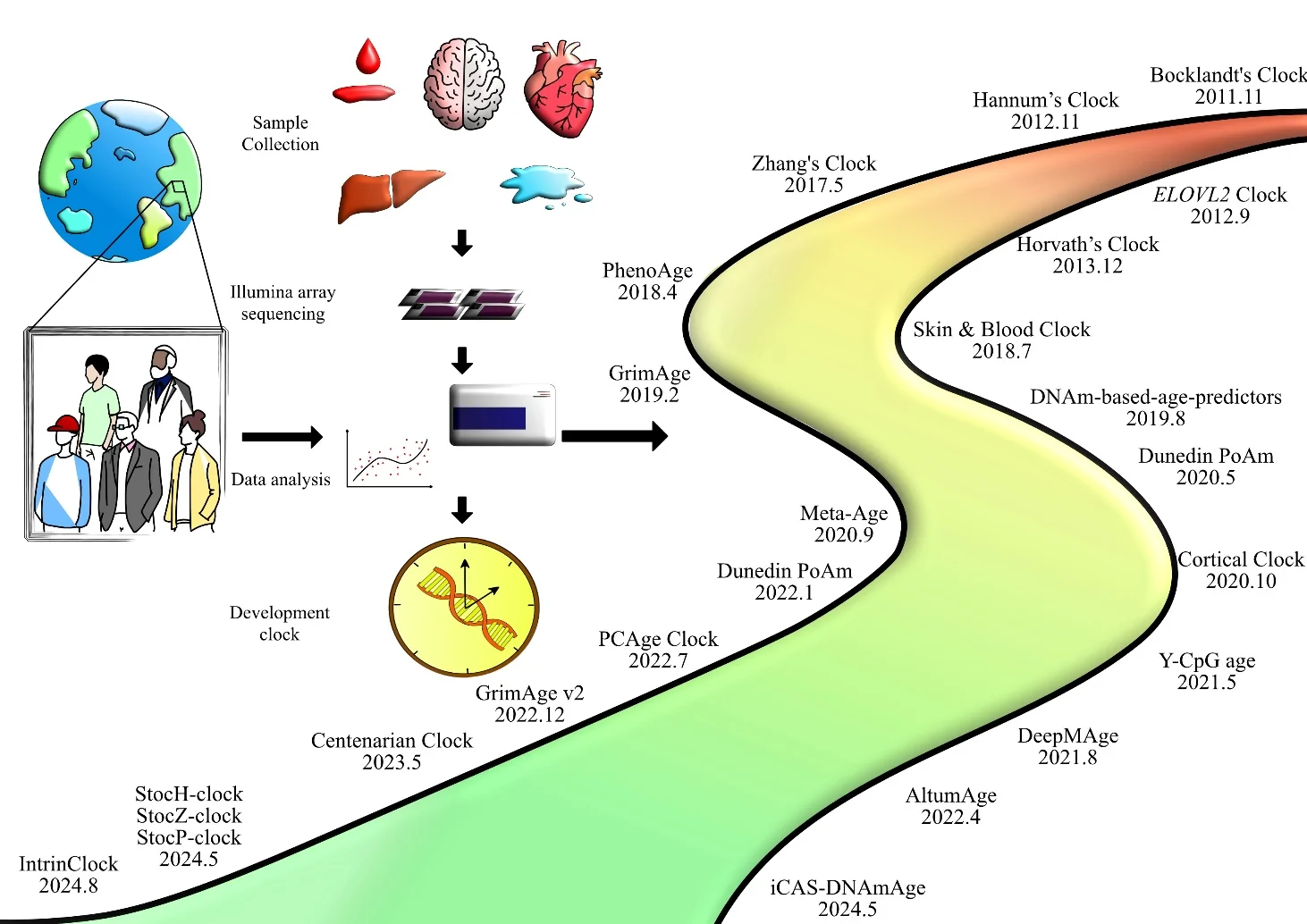
**The Development and Evolution of Epigenetic Clocks**. The image depicts the construction process of epigenetic clocks and outlines the approximate chronological evolution of their development. Typically, the construction process involves sample collection, DNA methylation site analysis using Illumina arrays, and the application of diverse data processing techniques to model and establish the clock.

In summary, epigenetic clocks, as promising biomarkers of biological age, have gained wide recognition for their sensitivity to age-related diseases, underscoring their value as vital tools in aging research. However, further refinement and integration are essential to enhance their applicability and reliability across various contexts, maximizing their potential to advance our understanding of aging and health [8, 27] (Fig. 2).

## 3. First-Generation Epigenetic Clocks

The initial models of epigenetic clocks used single-step regression to estimate biological age, using chronological age as a baseline for aging predictions. By analyzing methylation patterns at specific CpG sites, these models provide insights into biological age, thus often being referred to as “chronological clocks” [28]. First-generation epigenetic clocks demonstrated high accuracy in estimating chronological age, making them valuable tools for assessing biological aging. Discrepancies between predicted biological age and actual chronological age yield insights into the rate of aging, highlighting how genetic and environmental factors shape an individual’s physiological state.

### 3.1 Horvath’s Clock

A landmark model in epigenetic aging research, Horvath’s clock was the first to achieve cross-tissue age prediction by analyzing DNA methylation data from multiple tissue types. Although not the earliest epigenetic clock, its ability to use multi-organ, multi-tissue DNA methylation data marked a major advancement in cross-tissue aging analysis. Developed using publicly available datasets from 7,844 samples across 51 tissue and cell types on the Illumina 27K and Illumina 450K array platforms, Horvath’s clock employs 353 CpG sites—193 positively and 160 negatively correlated with age—to estimate epigenetic age [29].

The core strength of the Horvath clock lies in its high accuracy and broad applicability across diverse tissues and organs. It has been validated in almost all tissues and organs, including whole blood, brain, kidney, and liver, showing minimal age-related variance [14, 16, 30]. Research has also confirmed its effectiveness in aging [31] and age-related diseases [32], cancer [33], as well as in predicting lifestyle impacts and mortality rates [34]. Another significant advantage of the Horvath clock is its versatility; it can be applied not only to aging research in other mammals but also to in vitro aging analyses, underscoring its robustness across different tissues and experimental conditions [35]. Thanks to the Horvath clock, many previously unresolved scientific questions now have a relatively objective evaluation method, making it an invaluable tool for studying aging and related factors [30, 36]. Furthermore, as a pioneering work in epigenetic clocks, its significance extends to paving the way for the development of next-generation epigenetic clocks [37].

Horvath’s clock, though widely applicable, has specific limitations. As a “pan-tissue clock,” its predictive accuracy can vary across tissues, particularly in hormonally sensitive tissues and high-variability samples like blood [38]. Compared to newer models such as GrimAge and PhenoAge, Horvath’s clock demonstrates lower predictive consistency, potentially due to its capture of distinct biological pathways that complicate interpretation [34]. Environmental and genetic influences, including lifestyle factors like smoking and obesity, also affect its aging acceleration metrics, introducing heterogeneity into its predictions [16, 39]. Additionally, Horvath’s clock often underestimates biological age in individuals over 60, likely due to limited representation of older samples in its training dataset [40-42]. The clock exhibits limited sensitivity to certain diseases, proving unable to capture significant age acceleration in conditions like schizophrenia and progeroid syndromes [43]. Its sensitivity to specific age-related conditions, such as schizophrenia and progeroid syndromes, also remains limited. Improving cross-tissue accuracy, predictive consistency, and disease sensitivity could further enhance its utility, particularly through integration with other models [34].

### 3.2 Hannum’s Clock

Hannum’s clock is among the most prominent first-generation epigenetic clocks, alongside Horvath’s Clock. Although it was also developed using the Illumina 450K methylation array, Hannum’s clock stands out as one of the earliest epigenetic models specifically tailored to blood samples. This model was built upon over 450,000 CpG markers derived from whole blood samples of 426 Caucasian and 230 Hispanic adults, aged 19 to 101. Ultimately, 71 CpG sites with the strongest age-related changes were selected to estimate biological age [16, 44]. Developed using the Elastic Net algorithm—a technique that combines the benefits of ridge regression and LASSO regression—Hannum’s clock demonstrates a high correlation of 0.96 between biological and chronological age, with an average absolute error of 3.9 years (slightly higher than the Horvath clock’s 3.6 years) [44]. Optimized specifically for blood samples, Hannum’s clock shows greater specificity in studies concerning blood-based health and disease. Beyond estimating the gap between biological and chronological age, it boasts a strong association with clinical markers, making it a valuable tool for assessing the risk of various age-related diseases. Research has linked Hannum’s clock to indicators such as body mass index (BMI), cardiovascular health, immune function, and chronic conditions [16]. Its utility extends further to evaluating the impact of clinical interventions. By tracking changes in biological age before and after interventions—such as weight loss programs or exercise therapy—Hannum’s clock offers a quantitative measure of treatment outcomes, thus aiding in personalized health management [45, 46]. In addition, Hannum’s clock has shown sensitivity to psychological trauma, with studies demonstrating that accelerated aging, as measured by this clock in patients with post-traumatic stress disorder, correlates with the severity of their condition [47, 48].

In contrast, Hannum’s clock is limited in its applicability to tissues other than blood. Compared to the Horvath clock, Hannum’s clock exhibits lower sensitivity to external factors and reduced cross-ethnic adaptability [49]. Like other first-generation epigenetic clocks, it is based on static CpG sites and therefore unable to capture the dynamic aspects of aging, rendering it less effective at accurately reflecting the rate of aging [35, 37, 50]. Focusing solely on CpG sites also means that first-generation clocks, including Hannum’s, may overlook CpG methylation influences specific to certain cell types, such as neurons, and disregard the effects of various external factors on CpG methylation levels [51, 52]. Another critical limitation is their inability to account for epigenetic age acceleration caused by specific diseases, such as cancer. These shortcomings are expected to be addressed in second-generation epigenetic clocks through the incorporation of new biomarkers and indicators [33, 53].

### 3.3 Bocklandt Clock

The Bocklandt clock, developed in 2011, was the first epigenetic clock and introduced the groundbreaking hypothesis that methylation status at specific DNA sites changes in a predictable manner with age, showcasing the potential of DNA methylation for age prediction. Using the Illumina HumanMethylation27 microarray, this clock quantified the methylation levels of 27,578 CpG sites at single-nucleotide resolution in saliva samples from 34 pairs of monozygotic twins aged 21 to 55. Through linear regression modeling, Bocklandt’s team ultimately identified 88 CpG sites for age estimation [54]. That same year, Koch and colleagues aimed to expand the applicability of age prediction models by introducing an epigenetic aging signature composed of five CpG sites to estimate donor age across a broader range of tissues. However, these models exhibited a considerable margin of error from actual age, with an average absolute error of approximately 11 years, highlighting the need for further research to develop more precise clocks [55].

Although the Bocklandt clock may not enjoy the same level of recognition as Horvath’s Clock, it made groundbreaking contributions by first establishing the viability of using DNA methylation as a means for age estimation and by introducing an alternative approach to studying aging. This early development ignited a wave of research exploring the intricate relationship between DNA methylation and the aging process, thereby strengthening the role of epigenetic within aging research [56]. While its predictive accuracy and tissue specificity were limited, the Bocklandt clock marked a pivotal initial step in the evolution of epigenetic clocks [56, 57]. Ultimately, its true significance lies in the innovative connection it forged between epigenetic and aging, as well as in providing a novel framework that has guided subsequent research, driving forward the integration of aging studies and epigenetic science.

### 3.4 Skin & Blood Clock

The Skin & Blood clock, developed by Steve Horvath’s team, was specifically designed to capture the unique epigenetic characteristics of skin and blood tissues. Studies have shown that DNA methylation patterns in tissues such as skin and blood exhibit distinct dynamics compared to other tissues, making it advantageous to create dedicated clocks to improve predictive accuracy for these specific tissues [58, 59]. The construction of the Skin & Blood clock involved analyzing skin and blood samples from individuals across a wide age range, using data obtained from the Illumina 450K methylation array. Through this analysis, the researchers identified 391 CpG sites closely associated with age [60].

This extensive dataset, encompassing a broad range of ages, was chosen to ensure the clock’s applicability across diverse populations. The primary objective of the Skin & Blood clock is to enhance predictive precision within skin and blood tissues, thereby enabling more accurate assessments related to skin aging, immune system changes, and associated health conditions. By tailoring the clock to specific tissues, the Skin & Blood clock provides a more reliable tool for studying skin aging, biological age acceleration, and blood health. Horvath’s team has also developed an online DNA methylation age calculator for public use, available at “https://dnamage.genetics.ucla.edu/”.

### 3.5 DNAm-based-age-predictors

First-generation epigenetic clocks are primarily centered around age prediction, utilizing the difference between predicted and actual age as a biomarker of aging to assess biological aging rates. Nevertheless, these clocks, to varying degrees, are constrained by their training datasets and still exhibit some degree of error in age estimation. To address this, Zhang and colleagues investigated the theoretical possibility of an ideal DNA methylation-based age predictor. They collected 14 datasets, including blood and saliva samples, covering an age range from 2 to 104 years [61]. This clock demonstrated minimal influence from disease and showed stronger correlations in samples from blood, saliva, and endometrial tissues, although correlations were lower in brain samples [61, 62].

## 4. Second-Generation Epigenetic Clocks

Compared to first-generation epigenetic clocks, second-generation clocks have a somewhat different focus. While first-generation clocks primarily aim to predict chronological age, second-generation clocks place greater emphasis on integrating assessments of health status, disease risk, and aging rate. The distinction between these generations lies in the core objectives of the models, the number and characteristics of selected CpG sites, the inclusion of health and physiological factors, and the incorporation of dynamic aging rates [52].

Thus, second-generation epigenetic clocks are no longer limited to age-related CpG sites alone; they also account for an individual’s health status, disease risks, lifestyle factors, and other variables [63]. This approach provides a more comprehensive view of an individual’s biological state, allowing for a nuanced evaluation of age in relation to health, disease, and lifestyle [37, 64]. That said, clocks that incorporate a broader range of biological information tend to have reduced accuracy in pure age prediction [18, 65-67].

### 4.1 PhenoAge

Chronological age alone fails to fully encapsulate an individual’s health status and aging trajectory. To overcome the limitations of first-generation epigenetic clocks, Morgan Levine and colleagues developed an advanced epigenetic clock aimed at more accurately predicting health status and mortality risk by integrating DNA methylation data with diverse physiological markers [27]. Leveraging data from NHANES III, they initially selected nine clinical biomarkers to construct the “Phenotypic Age” model. By correlating Phenotypic Age with blood DNA methylation profiles, they developed DNAm PhenoAge, an epigenetic metric based on 513 CpG sites identified through Illumina 27K, 450K, and 850K arrays.

A distinguishing feature of the PhenoAge clock is its integration of DNA methylation data with nine key clinical biomarkers: albumin, creatinine, glucose, C-reactive protein, lymphocyte percentage, mean cell volume, red cell distribution width, alkaline phosphatase, and white blood cell count. This combination enhances its sensitivity to individual variations and refines the accuracy of biological age estimation [37]. The PhenoAge clock can capture age-related shifts tied to chronic diseases (including cardiovascular disease, diabetes, and cognitive decline) and all-cause mortality, establishing it as a powerful tool for predicting healthspan [27, 68]. PhenoAge also correlates with lifestyle and demographic factors, such as educational attainment, physical activity, income, systolic blood pressure, body mass index, dietary habits (as indicated by carotenoid levels), and smoking status[69]. In contrast, DNA methylation age acceleration derived from multi-tissue clocks shows weaker associations with lifestyle factors and inflammatory markers, indicating it may reflect a more genetically programmed, intrinsic aging process [14, 70].

Recent studies have employed the PhenoAge clock to assess the impact of targeted treatments, including pharmacological and lifestyle interventions, on biological aging. PhenoAge has shown promise in determining whether specific interventions can effectively decelerate biological aging, supporting the development of personalized health management strategies and informing clinical decision-making [71, 72]. Insights from the PhenoAge clock reveal that individuals with an accelerated epigenetic age frequently exhibit heightened pro-inflammatory and immune responses, as well as impaired cellular maintenance and repair functions. These findings underscore the link between epigenetic age acceleration and fundamental shifts in biological function [27].

As a second-generation clock, the PhenoAge model represents a marked advancement, with its inclusion of clinical biomarkers enhancing both predictive precision and relevance, underscoring the significance of these factors in modulating epigenetic age. This approach offers valuable insights for future clock development, suggesting that training clocks on an expanded set of impactful biomarkers could produce even more accurate aging models [28, 61]. Overall, the PhenoAge clock is a substantial enhancement over first-generation epigenetic clocks, offering a multidimensional measure of aging. However, its complexity poses challenges for large-scale population studies, and it remains highly responsive to environmental and individual variability. Further refinement is essential to reinforce its associations with specific disease risks [27, 37, 41, 73].

### 4.2 GrimAge

GrimAge is a comprehensive epigenetic clock developed in recent years, based on the idea that certain external factors (such as smoking) and internal protein biomarkers (such as inflammation-related proteins) have a greater impact on individual health and mortality risk than chronological age alone. Similar to other epigenetic clocks, GrimAge was trained on CpG sites shared by the Illumina Infinium 450K and Illumina EPIC methylation arrays [74]. The final model includes 1,030 CpG sites that optimally predict composite biomarkers of seven DNA methylation proteins, as well as annual smoking pack-years. Its defining feature is the integration of multiple DNA methylation markers and clinical biomarkers related to aging, such as smoking status, insulin resistance, and inflammation markers. Compared to other epigenetic clocks and biological age estimates, GrimAge provides more accurate predictions of all-cause mortality and has thus been referred to as the “death clock” [41].

To further enhance GrimAge, the team developed an updated version known as GrimAge version 2, or AgeAccelGrim [75]. This second version employs two elastic net regression models to reselect, calibrate, and adjust the weights of protein biomarkers associated with health and mortality risk. As a result, AgeAccelGrim demonstrates greater robustness, outperforming the original GrimAge in predicting mortality risk, computed tomography data, cognitive assessments, and lifestyle factors, and is also applicable to saliva samples. Compared to other second-generation epigenetic clocks, GrimAge shows superior correlation with age-related physical function decline and clinical phenotypes, such as walking speed, grip strength, Fried frailty, polypharmacy, the Mini-Mental State Exam (MMSE), and the Montreal Cognitive Assessment (MOCA) [37, 76]. AgeAccelGrim has also demonstrated stronger associations and predictive power for a range of diseases, such as type 2 diabetes and various cancers, highlighting its potential as a robust predictor of disease risk [77, 78]. Due to its higher complexity and inclusion of mortality-related biomarkers, GrimAge is also more sensitive to biological outcomes in socioeconomically disadvantaged communities [79]. Furthermore, GrimAge acceleration has been observed in severe depression, supporting its applicability in mental health research. The association between AgeAccelGrim and the severity of PTSD further suggests its clinical utility for tracking the long-term impact of trauma-induced stress on morbidity and mortality risk [80-82]. GrimAge’s dependence on protein biomarkers suggests that it might be less sensitive to detecting short-term health fluctuations and may be vulnerable to interference from acute inflammation, infections, or other sudden health events [83].

### 4.3 DunedinPoAm

Among the epigenetic clocks discussed, capturing a methylation snapshot of an individual or group at a single time point remains a commonly employed approach. Yet, to identify regions where methylation shifts due to aging or disease—or to predict future changes—a longitudinal study design is essential [28]. Longitudinal studies of this nature allow researchers to track methylation trends within individuals over extended periods, providing a more accurate view of dynamic epigenetic changes. Originating from the Dunedin longitudinal study, the DunedinPoAm (Pace of Aging Methylation, PoAm) offers insights beyond biological age by quantifying an individual’s “pace of aging” across defined time intervals [84]. Given its foundation in longitudinal methylation data, DunedinPoAm could be regarded as a third-generation epigenetic clock. This model tracks longitudinal changes across 18 biomarkers associated with the functional health of blood and various organ systems, using Illumina 450K and EPIC array data from individuals of the same age cohort. Distinct from earlier DNA methylation clocks, which estimate biological age retrospectively, DunedinPoAm measures the rate of aging, providing an immediate indicator of aging velocity rather than a cumulative aging estimate. Conceived as a “speedometer,” DunedinPoAm is highly sensitive to fluctuations in physical function, cognitive performance, motor skills, and visible markers of aging [84].

Research employing this clock has demonstrated that children exposed to adverse socioeconomic conditions early in life generally exhibit higher DunedinPoAm scores, while older adults with accelerated DunedinPoAm readings face an elevated risk of disease and mortality [85, 86]. Although these findings are valuable, the DunedinPoAm model is constructed from biological changes observed over a relatively short 12-year span, with data collected at only three time points. This constrained timeframe and limited sampling restrict its capacity to capture the complete aging trajectory throughout adulthood. The sparse data points also diminish its accuracy in assessing the rate of aging, which limits its effectiveness in clinical trials aimed at evaluating individual changes before and after treatment [87, 88]. To address these limitations, DunedinPACE was introduced as an improved version of DunedinPoAm. DunedinPACE extends the follow-up period, increases the number of assessments, and improves data reliability, resulting in a stronger correlation with health outcomes [88, 89]. Consequently, DunedinPACE represents a valuable complementary tool, offering useful advantages for quantifying biological aging through DNA methylation.

### 4.4 Zhang's clocks

Another clock that incorporates longitudinal analysis was developed by Zhang et al., based on a 14-year cohort study. This study ultimately selected 10 CpG sites from whole blood samples using the Illumina 450K array. These CpG sites are strongly associated with all-cause mortality, cardiovascular disease, and cancer mortality, and they differ from those used in other current clocks [90]. While the Zhang clock demonstrates high predictive accuracy in mortality due to its specific focus, it is less effective than more complex clocks at capturing multidimensional health and aging characteristics or assessing risk for specific diseases [35, 91].

### 4.5 Principal component clock

As research advances, we are uncovering not only the links between epigenetic age, aging processes, and associated risk factors, but also the capacity of certain interventions to modulate epigenetic age [16, 92]. Yet, it is crucial to acknowledge the potential influence of technical noise in DNA methylation measurements. Studies have shown that, beyond issues inherent to sample preparation and hybridization, the presence of unreliable and poorly reproducible probes introduces substantial challenges in the accurate measurement of methylation at numerous CpG sites on methylation arrays—a persistent issue in both Illumina 450K and EPIC platforms [93-95]. With the continued accumulation of data, this decline in measurement reliability and reproducibility could undermine the accuracy of research findings and impede the construction of precise clocks, particularly in first-generation models where heterogeneity is notably high [87, 96, 97]. The difficulty of disentangling biological variation from technical noise poses a significant challenge, as errors in epigenetic clocks may become progressively magnified. This issue becomes especially concerning in longitudinal studies assessing intervention effects, where the repeated amplification of noise could ultimately compromise the reliability of the findings.

To mitigate these issues, Higgins-Chen et al. developed a computational solution to enhance the robustness of epigenetic clocks, utilizing principal component (PC) analysis to retrain several prior clocks, including Horvath, Hannum, and GrimAge clocks. This approach allows for more stable results with smaller sample sizes and has been shown to be particularly reliable and effective in tissues such as saliva and brain [23]. In clock construction, PC analysis is a highly effective training method; it can address the “curse of dimensionality” that arises as the number of features in methylation data expands with sample size, reducing risks of overfitting and multicollinearity, thereby enhancing model performance [98].

In this context, Fong et al. identified features that differentiate healthy and unhealthy aging trajectories, resulting in the development of the clinical aging clock PCAge and a simplified aging clock, LinAge. They also demonstrated a method for customizing clinical clocks for specific datasets by retraining a tailored clock model [99]. PCA-trained clocks, such as PCAges, increase the average epigenetic age of samples, aligning them more closely with chronological age. Furthermore, PCA-based measurement methods have been shown to better predict aging-related health outcomes, including mortality, without altering the original relationships between health behaviors and outcomes considered in the initial clock models [62, 93].

## 5. Other Clocks

Beyond the well-studied classic clocks described above, a variety of other epigenetic clocks have emerged, showcasing researchers’ innovative approaches to bridging gaps in current aging research. While these clocks have not yet been extensively validated or rigorously evaluated, their development has greatly enriched the field, advancing our understanding of biological aging. Notably, this category encompasses clocks that fall outside the traditional classifications of first- and second-generation epigenetic clocks.

### 5.1 Centenarian Clocks

First-generation epigenetic clocks tend to significantly underestimate the ages of centenarians due to their reliance on regression toward the mean. To address this, Eric Dec and colleagues developed Centenarian clocks, specifically designed to provide accurate age estimations for individuals over 100 years old. This innovation is expected to aid in verifying hypotheses related to exceptional longevity [100].

### 5.2 ELOVL2 Clock

The development of aging biomarkers has been a challenging and protracted process, with the identification of age-related biological factors remaining a key objective in aging research. *ELOVL2* has emerged as a promising biomarker of aging due to its consistent increase in methylation from the earliest stages of life, playing a significant role in many age-related molecular mechanisms [101, 102]. Garagnani et al. observed that methylation levels of *ELOVL2* in whole blood DNA samples showed a striking, almost “on-off” pattern, increasing from 7% to 91% with age [103]. With an approximate prediction error of 5.5 years, the *ELOVL2* clock demonstrates considerable accuracy, positioning it as a promising molecular tool for forensic age estimation [104, 105]. While this clock shows significant potential, numerous models centered on *ELOVL2* have been developed, underscoring the need for further validation to clarify its broader applications in aging research [106].

### 5.3 Cortical Clock

It is well-known that the aging process contributes to a range of age-related diseases, among which neurodegenerative disorders severely impact the quality of life in later years. DNA methylation changes in the cortex are closely associated with neurodegenerative diseases such as Alzheimer’s and Parkinson’s [107, 108]. To address the limitations of earlier clocks—which were often not rigorously calibrated for specific tissues and were prone to age-related phenotypic influences leading to false positives—recent efforts have focused on developing clocks specifically for the human cerebral cortex [109, 110]. These cortical clocks weigh the DNA methylation levels at specific sites, distinguishing themselves from clocks developed from multi-tissue or blood samples. They excel in predicting cortical age and are sensitive to neurodegenerative diseases and common aging phenotypes [30]. However, like other specialized clocks, cortical clocks lose their predictive accuracy when applied to tissues outside their intended target, limiting their cross-tissue applicability [61].

### 5.4 Meta-clock

In light of the heterogeneity and overlapping signals among existing clocks, Liu and colleagues combined multi-omics data from various human tissues and cell types, integrating findings from in vitro experiments focused on aging markers. This approach provided a comprehensive view of the shared and contrasting features captured by 11 existing epigenetic clocks. By synthesizing the conserved features across these clocks, they developed a new clock, termed the “Meta-clock.” This clock aims to combine the best aspects of previous clocks, demonstrating higher accuracy in mortality prediction and greater robustness in capturing aging-related changes both in vivo and in vitro [35].

### 5.5 iCAS-DNAmAge

Variability in clock construction can also arise from individual inconsistencies, which are further magnified across different cohorts, potentially impacting results. Significant differences in epigenetic age have been observed across racial and ethnic groups [93, 111, 112]. This raises uncertainty as to whether existing epigenetic clocks can accurately capture aging in cohorts that differ racially or ethnically from the populations on which these clocks were trained. To address this issue, Zheng and colleagues developed a new clock called iCAS-DNAmAge, along with a multimodal aging predictor [113]. This clock was built using data from a Chinese cohort, allowing for more accurate age estimations for Chinese individuals compared to previous cohort-based clocks. A major limitation of current epigenetic clock research is its heavy reliance on data from European ancestry cohorts, resulting in a lack of representation from other populations and insufficient consideration of genetic heterogeneity [22]. Large-scale, longitudinal studies across diverse populations are essential for expanding the applicability of epigenetic clocks [114]. The iCAS-DNAmAge clock contributes to bridging this gap, enhancing the utility of epigenetic clocks across different regions and ethnicities.

### 5.6 Y-CpG Age

Extensive research has demonstrated that women tend to outlive men, with epigenetic factors emerging as a potential underlying mechanism for this difference in lifespan. Epigenetic clocks, which are biomarkers of biological age, reveal distinct sex-based variations. These differences encompass a range of epigenetic features, including methylation patterns on the X chromosome, X-chromosome inactivation, histone modifications, hormonal regulation, and DNA methylation profiles that are specific to each sex [115-118]. Additionally, the expression of certain epigenetic regulators on sex chromosomes appears to play a unique role in modulating methylation dynamics, influencing how age-related changes are expressed differently in men and women [119, 120].

Further studies on age estimators specifically targeting the Y chromosome have revealed a progressive increase in hypermethylation of Y-linked CpG sites as men age[119, 121]. These Y chromosome-specific epigenetic clocks exhibit high predictive accuracy, particularly in individuals over the age of 40, suggesting their potential utility in forensic and criminological applications where age estimation may be critical. Furthermore, the development of sex chromosome-specific epigenetic clocks holds promise for deepening our understanding of the interplay between longevity and gender. Through investigating epigenetic mechanisms that may contribute to the female longevity advantage, these specialized clocks hold the potential to reveal insights into the relationship between longevity and gender from an epigenetic perspective.

### 5.7 Neural Network-Based Clocks

Artificial intelligence has shown remarkable advantages in prediction and diagnosis across various fields [122]. Neural networks, a core component of deep learning, have been a driving force behind the rapid advancements in modern AI. Traditional epigenetic clocks mostly rely on regression methods, whereas DeepMAge was developed using neural networks with blood samples as the training set, demonstrating superior performance compared to conventional clocks [123]. DeepMAge has a median absolute error of around 2.77 years in age prediction, though certain diseases can impact its accuracy. This limitation may stem from a lack of training data from other tissues and limited longitudinal data.

AltumAge addresses some of these shortcomings. Trained on samples from 142 different experiments, it can, like the Horvath clock, be used to estimate pan-tissue age. Unlike linear clocks, AltumAge excels in accuracy, applicability, robustness, and resistance to interference, highlighting the advantages of neural network-based models over linear approaches [124]. AltumAge is highly sensitive to various diseases and biologically relevant conditions, including cancer, type 2 diabetes, HIV, obesity, anxiety, and smoking [124-126]. AltumAge also possesses the capability to capture age-related interactions between CpG sites, with samples from tumors, immune dysfunction, and mitochondrial dysfunction displaying elevated predicted ages. In general, this clock outperforms traditional linear regression models across various performance metrics, while simultaneously providing unique biological insights into the mechanisms of aging. [124, 127].

### 5.8 Epigenetic Clocks for Model Organisms

Model organisms are an indispensable part of aging research. Similar to humans, DNA methylation data has revealed abundant age-related markers in various model organisms, leading to the development of species-specific epigenetic clocks. Some clocks developed for rodents, for example, exhibit characteristics similar to human epigenetic aging markers [128, 129]. Lu and colleagues sought to construct a universal pan-mammalian epigenetic clock, proposing three pan-mammalian age estimators that provide both absolute and relative age measurements with more reliable biological features, thereby improving accuracy and versatility across species [130]. For chimpanzees, a species-specific epigenetic clock has highlighted significant overlap in age-related epigenetic patterns between chimpanzees and humans, offering new insights for comparative aging research [131]. Clocks have also been developed for invertebrates and non-mammalian species, including fruit flies and nematodes, thereby enabling aging research across a broader range of species and in vitro models. These advancements expand the toolkit available for exploring the intricate relationship between epigenetic and aging, providing valuable insights into conserved mechanisms across diverse biological systems. [132-134].

Beyond the primary epigenetic clocks discussed above, other models have emerged in recent years. The development of these emerging clocks demonstrates the potential applications of epigenetic clocks in diverse biological and clinical contexts. This includes clocks developed using new technologies, those tailored to specific biological aspects, and some that represent corrections or updates to previous clocks [24, 66, 135]. There are also clocks with unique advantages that are not included here due to limited information, lack of open-source data, or insufficiently distinguished performance characteristics. Further research is needed to explore the applicability and generalizability of some of these clocks [21, 136]. A brief summary of the key characteristics of human epigenetic clocks can be found in Table 1.

**Table 1:** Summary of Key Characteristics of Human Epigenetic Clocks.

Clock	Data Source	Final CpG number	Training Method
First-generation epigenetic clock			
Horvath's Clock [29]	Multi-tissue Illumina 27K / 450K	353	Elastic net regression
Hannum's Clock [44]	Whole Blood Illumina 450K	71	Elastic net regression
Bocklandt's Clock [54]	Saliva Illumina 27K	88	multivariate linear regression
Skin & Blood Clock [60]	Skin and Blood Illumina 450K	391	Elastic net regression
DNAm-based-age-predictors [61]	Whole Blood and Saliva Illumina 450K/EPIC	514	Elastic Net and Best Linear Unbiased Prediction

Second-generation epigenetic clock			
PhenoAge [27]	Whole Blood Illumina 27K / 450K	513	Cox regression with penalized elastic net
GrimAge [74]	Whole Blood Illumina 450K / EPIC	1030	Cox regression with elastic net
GrimAge v2 (AgeAccelGrim) [75]	Whole Blood Illumina 450K / EPIC	1030	Cox regression with penalized elastic net
DunedinPoAm [84]	Dunedin Study Illumina 450K/EPIC	46	Elastic net regression
DunedinPACE [88]	Dunedin Study Illumina 450K/EPIC	173	Elastic net regression
Zhang's Clock [90]	ESTHER study Whole Blood and Saliva Illumina 450K	10	LASSO regression
Principal Component Clock (PCAge) [23]	Whole Blood Illumina 450K/EPIC	78464	PCA with Elastic net regression

Other epigenetic clocks			
Centenarian Clocks [100]	Whole Blood and Saliva Illumina 450K/EPIC	33495	Elastic net regression and neural network models
*ELOVL2* Clock [103, 104]	Whole Blood Illumina 450K	2	Linear regression model
Cortica Clock [110]	Cortical Illumina 450K	347	Elastic net regression
Meta-clock [35]	Framingham Heart Study Multi-tissue Illumina 450k	878	Elastic net regression
iCAS-DNAmAge [113]	Whole Blood Illumina EPIC/EPICv2	65	Elastic net regression
DeepMAge [123]	Whole Blood Illumina 27K / 450K	1000	Deep neural network
AltumAge [124]	Multi-tissue	20318	Deep neural network
StocH-clock [66]	Whole Blood and sorted immune cells Illumina 450k	353 based on Horvath’s clock	Elastic net regression with stochastic simulation modeling
StocZ-clock [66]	Whole Blood and sorted immune cells Illumina 450k	514 based on Zhang’s clock	Elastic net regression with stochastic simulation modeling
StocP-clock [66]	Whole Blood and sorted immune cells Illumina 450k	513 based on PhenoAge clock	Elastic net regression with stochastic simulation modeling
IntrinClock [137]	Whole Blood Illumina 450K/EPIC	381	Elastic net regression
Y-CpG Age [121]	Male Y chromosome Illumina 450K	75	Linear regression model
CausAge[138]	Whole Blood Illumina 450K	586	Elastic net regression
AdaptAge[138]		1000	
DamAge[138]		1090	

## 6. Using the Past to Predict the Present and Future

DNA methylation encapsulates an individual’s formative experiences and accumulated life history. During embryogenesis, DNA methylation undergoes a comprehensive reset, ensuring that the genome of the next generation begins from a “clean slate.” From that point forward, each experience—whether dietary, emotional, environmental, or disease-related—leaves distinct, cumulative marks on this genomic slate. This accumulation of influences shapes DNA methylation patterns, enabling it to serve as a partial chronicle of one’s life history [139]. Unlike histone or RNA methylation, DNA methylation exhibits notable stability, functioning as a persistent regulatory mechanism for gene expression, while also retaining the capacity to respond dynamically and reversibly to short-term stimuli [140, 141]. These methylation patterns display remarkable consistency across diverse tissues, positioning DNA methylation as an ideal systemic biomarker [59, 142]. This “genomic memory” effectively archives cellular experiences over time, which epigenetic clocks strive to decode to infer current biological states and project future health outcomes.

**Table 2:** Summary of Key Characteristics of Human Epigenetic Clocks

Clock	Training Dataset	Advantages	Limitations	Application
First-generation epigenetic clock				
Horvath's Clock [29]	Based on over 8,000 samples from 51 healthy tissues, including blood, brain, liver, and skin.	High accuracy for age prediction in multiple tissues; first multi-tissue epigenetic clock enabling comparisons across different cell types, including iPS and embryonic stem cells.	May underestimate biological age in older adults, lacks sensitivity to certain diseases, and exhibits variability in tissues such as breast and skeletal muscle. Highly sensitive to environmental factors.	Designed to deliver consistent age predictions across various tissues by analyzing methylation levels, offering insights into age acceleration in disease contexts like cancer.
Hannum's Clock [44]	Based on blood samples from 656 individuals aged 19 to 101; primarily Caucasian and Hispanic individuals.	Demonstrates high prediction accuracy for age in blood	Limited to blood tissues; lower cross-ethnic adaptability; static CpG sites fail to capture dynamic aging processes.	Exhibits strong specificity for blood-related studies and demonstrates robust associations with clinical biomarkers, incorporating analyses of sex and genetic variations.
Bocklandt's Clock [54]	Developed from saliva samples of 34 identical male twin pairs (ages 21-55) and validated on additional 60 independent samples (31 males, 29 females, ages 18-70).	Offers accurate age predictions from saliva samples, with potential forensic applications and relevance for assessing age-related health risks.	Suffers from large prediction errors, with restricted tissue specificity and limited accuracy. Currently applicable only to saliva samples.	Potential tool for forensic age estimation; may aid in clinical assessments by measuring biological age and associated health risks.
Skin & Blood Clock [60]	Trained on data from blood, skin, and fibroblast samples using data from Illumina 450K, covering ages from infancy to elderly samples.	Accurate age estimation in skin, blood, and saliva; ideal for forensic applications and ex vivo studies; accurately tracks age acceleration in conditions like Hutchinson-Gilford Progeria Syndrome.	Predictive accuracy declines in non-skin and non-blood tissues, with slight variability observed in fibroblast measurements, particularly in very young samples. Its applicability is limited to target tissues.	Useful for biomedical applications including ex vivo aging assays for screening anti-aging compounds and forensic age estimations in skin and blood samples.
DNAm-based-age-predictors [61]	Involves multiple cohorts, such as LBC1921, LBC1936, and Generation Scotland, spanning over 13,000 blood samples and additional samples from saliva, brain, and endometrium.	High prediction accuracy with increasing training size; offers insights on age-associated changes across tissues, enhancing biological age assessment.	Performance varies across tissue types, showing reduced effectiveness in smaller sample sizes and age variance influenced by sample types (e.g., blood vs. saliva). Correlation is particularly low in brain tissues.	Promising for refining biological age prediction in clinical applications, with potential to identify health trajectories across diverse tissue samples.

Second-generation epigenetic clock				
PhenoAge [27]	Based on whole blood samples from the National Health and Nutrition Examination Survey (NHANES III and IV) and validated across multiple cohorts, including InCHIANTI and Framingham.	High predictive power for morbidity and mortality, with stronger associations to health-related aging outcomes (e.g., all-cause mortality, physical functioning) than chronological age estimators.	Characterized by high complexity and resource demands, making it unsuitable for large-scale population studies.	Useful for tracking biological health and assessing aging-related disease risks, outperforming traditional clocks by emphasizing healthspan and lifespan prediction over simple chronological age.
GrimAge [74]	Trained on data from the Framingham Heart Study (FHS) with 2,356 whole-blood samples; validated across over 7,000 samples from independent cohorts like WHI, InCHIANTI, and JHS.	Strongly predictive of lifespan and healthspan; significant associations with mortality risk, time-to-disease events (e.g., coronary heart disease, cancer), and lifestyle factors.	While accurate in predicting 12 plasma proteins, its reliability for other proteins remains low. Further validation is needed for populations beyond European, African American, and Hispanic groups. Sensitive to acute inflammation and prone to interference from sudden health events.	Valuable for lifespan and health monitoring in clinical settings, including human anti-aging studies and age-related disease prevention, serving as a complementary tool to existing clinical biomarkers.
GrimAge v2 (AgeAccelGrim) [75]	Based on Framingham Heart Study with 1,833 samples for training and 711 for testing; validated across nine cohorts totaling 13,399 blood samples spanning European, African, and Hispanic populations.	Strong predictor of mortality, chronic diseases (e.g., type 2 diabetes), and conditions like coronary heart disease; tracks age-related healthspan decline across diverse racial/ethnic groups and biomarkers.	Primarily validated in blood samples; further research needed for effectiveness in non-blood tissues and greater ethnic diversity outside the current sample composition.	Useful in clinical settings for healthspan monitoring, offering early detection for age-related conditions and supporting intervention effectiveness in geroscience trials.
DunedinPoAm [84]	Based on longitudinal DNA methylation data from the Dunedin Study, tracking whole-blood samples and changes in 18 biomarkers from individuals born in 1972-1973 at ages 26, 32, and 38.	Assesses biological aging pace, correlates with physical/cognitive aging, lifestyle factors, chronic diseases, and mortality.	Primarily validated in European populations, requiring additional testing in non-European groups and across diverse tissue types. Insufficient longitudinal data points make it challenging to capture the full trajectory of aging across adulthood.	Ideal for assessing age-slowing interventions in clinical trials and monitoring health span, especially in personalized medicine, by quantifying biological aging rates instead of static age.
DunedinPACE [88]	Expanded the Dunedin cohort dataset to include 20 years of data, incorporating a fourth measurement point at age 45.	Offers longer follow-up, more assessments, and higher reliability compared to DunedinPoAm.	Limited by training dataset diversity; improvements for non-European populations and external samples remain minimal.	More reliable than DunedinPoAm; suitable for geroscience trials and as a healthspan indicator in clinical settings.
Zhang's Clock [90]	Developed using blood samples from 1,000 participants (ages 50-75) in the ESTHER study (case-cohort design) and validated in the KORA cohort, spanning a broader age range (31-82).	Strong predictor of all-cause mortality, with risk ratios of up to 7.36 times for participants with high risk scores (5+); informative for clinical risk stratification.	Limited to blood samples and primarily validated in European populations, with further validation required in other ethnic groups. Demonstrates weaker associations with health traits compared to more complex clocks.	Valuable for risk assessment and personalized health monitoring, with potential for clinical integration in mortality risk stratification by focusing on disease-associated methylation changes rather than chronological age.
Principal Component Clock (PCAge) [23]	Trained on multiple DNA methylation datasets from the Framingham Heart Study, Health and Retirement Study, InCHIANTI, and various in vitro and in vivo samples, covering blood, saliva, and brain tissues.	Significantly improved reliability with 90% of replicate agreement within 1-1.5 years; effective in longitudinal and intervention studies for aging and personalized medicine.	Highly dependent on large datasets for PCA construction, with reduced effectiveness when sample variance is restricted, or batch effects occur across multiple datasets. Technical noise may still impact predictions.	Provides reliable tracking for age and health interventions, enabling reduced sample sizes in clinical trials; supports use in aging research across various tissues and health contexts.

Other epigenetic clocks				
Centenarian Clocks [100]	Developed using 7,039 samples from individuals aged over 40, including blood and saliva, with a notable cohort of 184 centenarians, 122 semi-supercentenarians (105+ years), and 25 supercentenarians (110+ years).	Demonstrates high accuracy in predicting age in individuals over 80, particularly well-suited for extreme ages (100+); validates claims of exceptional longevity.	Limited robustness when applied to ages beyond 115; current model might underestimate age for the very oldest-old due to regression-to-the-mean effects.	Useful in validating age claims for supercentenarians, supporting studies on longevity and possibly forensic investigations involving age estimation.
*ELOVL2* Clock [103, 104]	Initial study used 64 whole blood samples of various ages, followed by a larger validation on 501 samples aged 9 to 99 years, including cord blood.	High accuracy for chronological age prediction, robust in blood and bloodstain samples; maintains stability after weeks of storage at room temperature, valuable for forensic applications.	Limited applicability to non-blood samples; potential environmental influence on methylation stability for samples stored long-term.	A promising tool for forensic age estimation, particularly suited for determining the age of blood samples and bloodstains, with potential applications in forensic sciences and monitoring age-related health changes.
Cortica Clock [110]	Trained on 1,047 human cortical tissue samples with an additional 350 for testing, spanning ages 1 to 108 years.	High prediction accuracy in cortical tissue; minimizes error in age estimation for brain-specific aging studies, avoiding biases of traditional clocks used in brain tissue.	Limited applicability to non-brain tissues; model accuracy declines when applied to non-cortical samples such as blood.	Suitable for studying age-related brain conditions like Alzheimer’s and other neurodegenerative diseases, aiding in research on brain-specific aging processes.
Meta-clock [35]	Built on 2,993 samples from the Framingham Heart Study (FHS) across multiple tissues, including blood, brain, and skin.	High predictive accuracy for mortality (HR = 6.19); distinguishes tumor vs. normal tissues and tracks hallmarks of cellular aging like senescence and mitochondrial dysfunction.	Limited validation across different ethnic groups; some limitations in capturing aging signals specific to non-blood tissues.	High applicability in clinical aging studies, potential for healthspan and lifespan assessments, and disease risk monitoring.
iCAS-DNAmAge [113]	Based on 1,580 samples from two independent Chinese cohorts (Quzhou and CAS), covering age ranges from 20 to 87, all whole-blood samples.	High accuracy for biological age estimation, particularly adapted for Chinese populations; responsive to disease states and inflammation markers like IL-6.	Limited cross-ethnic validation, potential gender bias since initial testing included primarily female samples.	Suitable for broad health monitoring in Chinese populations, with potential expansion to track disease progression and interventions.
DeepMAge [123]	Derived from 4,930 blood samples across 17 studies; verified on 1,293 samples from 15 studies, all whole-blood samples.	High accuracy (MedAE = 2.77 years in verification); disease sensitivity (e.g., inflammatory bowel disease, multiple sclerosis); minimal sex bias in predictions.	Limited to blood samples; not tested in longitudinal settings; requires complex data preprocessing and model tuning.	A powerful tool for tracking age-related health changes, with superior sensitivity to diseases and improved age prediction over traditional clocks like Horvath’s.
AltumAge [124]	Trained on 8,999 samples from various tissues, enabling accurate cross-tissue age estimation.	Highly accurate in endometrial age prediction (MAE = 3.6 years), outperforming some first-generation epigenetic clock, with significant relevance to reproductive health.	Primarily trained on static samples; longitudinal stability in tissues with cyclic changes, like the endometrium, remains uncertain.	Potential tool for tracking age-related health changes,useful in assessing disease-related age acceleration.
StocH-clock [66]	Analyzed in 22,770 samples, including blood and sorted immune cell datasets from 25 independent cohorts (e.g., MESA, BLUEPRINT) to model the stochastic component in aging.	Quantifies the stochastic contribution in epigenetic aging, demonstrating that chronological aging prediction may rely largely on stochastic processes; provides insights into the variance explained by randomness.	Current models are primarily based on blood and immune cell data, requiring further validation for other tissue types and larger datasets to generalize findings.	StocH achieves R² ~0.66 in whole blood, StocZ shows higher stochastic accuracy for chronological age (~90% of Zhang's predictive accuracy), while StocP confirms non-stochastic influences on PhenoAge’s biological aging.
StocZ-clock [66]				
StocP-clock [66]				
IntrinClock [137]	Based on whole blood samples from the National Health and Nutrition Examination Survey (NHANES III and IV) and validated across multiple cohorts, including InCHIANTI and Framingham.	High predictive power for morbidity and mortality, with stronger associations to health-related aging outcomes (e.g., all-cause mortality, physical functioning) than chronological age estimators.	Primarily validated in blood samples; additional research needed for use in diverse tissues and ethnicities outside the original cohorts.	Useful for monitoring biological health and predicting aging-related disease risks, making it suitable for clinical aging assessments and health interventions.
Y-CpG Age [121]	Based on blood samples from 1,057 male individuals aged 15-87, collected from six different datasets focused on healthy individuals.	Provides the first male-specific age estimator, with applications in forensic age estimation for mixed samples and study of male-specific aging.	Currently limited to blood data; further studies are needed to validate effectiveness in other tissues and broader population samples.	Offers potential for forensic use in age prediction for male samples, and aids in the investigation of age-related changes specific to the Y-chromosome.
CausAge [138]	Developed novel epigenetic clocks utilizing putative causal CpG sites identified through EWMR, trained on a dataset of 2,664 blood samples.	Built using causally aging-associated sites, this clock delivers more stable age assessments compared to correlation-based clocks and is less susceptible to factors absent in the training data.	Shows slightly reduced performance in mortality prediction.	Exhibits higher accuracy and robustness in age prediction, forming the foundation for AdaptAge and DamAge development.
AdaptAge [138]	Causal CpG sites were further inferred and categorized into two distinct clocks based on the magnitude of Mendelian randomization causal effects and the direction of age-related differential methylation.	Unlike other clocks, the sites in this clock are either protective or neutral, with AdaptAge acceleration indicating enhanced healthspan.	Displays marginally lower predictive capacity in certain aspects compared to some second-generation clocks.	Significantly inversely correlated with mortality, it better reflects protective measures against aging and serves as a preferred biomarker for developmental events influencing aging traits.
DamAge [138]		Comprising exclusively damage-associated sites, this clock demonstrates higher correlations with damage rates and significantly outperforms other clocks.		Provides a more robust representation of age-related conditions, reliably reflecting age acceleration across various diseases. It is also a preferred biomarker for developmental events shaping aging traits.

Aging, in part, follows a stochastic course, with variability that becomes particularly pronounced among individuals [143, 144]. Paradoxically, this inherent stochasticity contributes to enhanced predictive accuracy. While the system is naturally disordered, the accumulation of random modifications across numerous genetic loci and cells leads to an averaged effect. This culminates in an emergent pattern that reflects a statistical regularity underlying the process. Consequently, these cumulative random changes produce a statistically consistent macro-scale pattern, which resembles a linearly increasing ‘marker of temporal progression [44, 145]. When stochastic accumulations exceed a certain threshold, they generate a robust aging signal, enabling epigenetic clocks to estimate age with remarkable accuracy, closely reflecting chronological age. This stable distribution aligns with the principles of entropy, where the gradual increase in entropy at the macro level follows a predictable trajectory [18, 146-148]. This phenomenon underscores the universality of epigenetic clocks, enabling them to sustain high predictive accuracy across diverse ages, individuals, and tissue types. The closer a clock’s estimates align with chronological age, the more likely it is capturing the aggregated effects of random DNA methylation alterations rather than specific biological pathways [66, 149].

In contrast, variations in biological age exhibit less randomness. DNA methylation changes driven by environmental and lifestyle factors—such as exposure to pollutants, socioeconomic status, and smoking—or by biological processes, including infections and immune system dynamics, are often governed by distinct mechanistic pathways rather than random variation [17, 79, 150]. These changes often follow discernible patterns and depend more on individual circumstances rather than maintaining a strictly linear correlation with time [66]. Furthermore, current epigenetic clocks are not confined to linear predictions. Although second-generation clocks demonstrate proficiency in estimating aging rate, mortality risk, and related aspects, refining these models to capture the complexities of aging mechanisms demands continued investigation. Aging remains a multifaceted and intricate process to model; while epigenetic clocks offer valuable insights into future aging trajectories, ongoing advancements are essential for greater precision.

## 7. Epigenetic Clocks as Tools for Measuring the Information Theory of Aging

The integration of epigenetic clocks with aging theories has unlocked promising avenues for understanding and measuring biological aging. By decoupling biological changes from the construct of chronological time, epigenetic clocks establish a distinct metric for assessing biological time. These clocks transcend simple age estimation; they reveal authentic differences in biological age and health status across individuals. Notably, epigenetic changes tied to aging are partially reversible—a dynamic that epigenetic clocks can also capture [151, 152]. Interventions such as lifestyle enhancement, stress reduction, and physical exercise have been shown to influence epigenetic age, effectively “rewinding” the clock and promoting a DNA methylation profile indicative of a more youthful state [153-155].

In line with the information theory of aging, biological information within an organism becomes increasingly disordered with age, reflecting a rise in entropy—a process seen as an intrinsic aspect of aging. The degradation and disruption of DNA methylation patterns exemplify this entropic shift [18, 156]. Rejuvenating the epigenetic age toward a younger state can be interpreted as a form of entropy reversal. Although aging generally follows a trajectory of increasing entropy, biological systems are not closed and isolated, which renders entropy reversal feasible [157]. Cellular reprogramming, particularly through the use of OSKM factors, exemplifies this potential; it enables the resetting of epigenetic information and extends cellular lifespan, observable through the reversal of epigenetic age [24, 56, 158, 159]. Thus, with targeted intervention strategies, biological age may be reset, with epigenetic clocks providing a concrete metric of this rejuvenation effect [92, 160].

Shannon entropy—originally a measure rooted in probability theory—has been adapted to analyze DNA methylation data, capturing the cumulative, age-related disorder across CpG sites over time [161]. By quantifying the probability distribution of methylation levels, Shannon entropy facilitates the identification of CpG sites with significant age correlations [18]. Compared to clocks reliant solely on methylation percentage metrics, entropy-based clocks may offer a more precise assessment of methylation age, potentially enhancing the accuracy of biological age estimation [162, 163].

## 8. Development and Challenges of Epigenetic Clocks

As tools for assessing epigenetic aging, epigenetic clocks have garnered increasing attention due to their broad biological relevance and potential to evaluate the effects of anti-aging interventions [64]. Most existing epigenetic clocks have been developed using data from Illumina methylation arrays, which offer low cost and reduce confounding factors associated with platform and technical variability, making these clocks accessible for computation by others [164, 165]. Calculations for various clocks based on Illumina data can be performed through Horvath’s online calculator at https://dnamage.genetics.ucla.edu/ and the “methylclock” R package [166].

Illumina array data, while widely used, come with notable limitations. Beyond issues like technical noise and low reproducibility due to problematic probes, some probes can bind to multiple genomic regions, potentially leading to false-positive results [167]. Furthermore, because the arrays target predefined CpG regions, they may overlook other potentially important methylation sites, such as non-CpG methylation and low-complexity regions. Microarray analysis measures each CpG site independently and cannot capture continuous DNA methylation patterns, which may limit the predictive accuracy of epigenetic clocks in specific tissues or under certain conditions [164, 168]. These limitations can lead to variability in results and reduced reliability [23].

Whole-genome bisulfite sequencing (WGBS), the gold standard for DNA methylation research, can cover about 90% of CpG sites, overcoming many limitations of Illumina arrays and offering superior resolution and localization accuracy to describe methylation states [169-171]. WGBS and reduced-representation bisulfite sequencing (RRBS) enable analysis of continuous methylation patterns, methylation haplotype blocks, and methylation heterogeneity. Standardizing sequencing depth and library preparation can reduce inter-sample variability [135, 169].

Thus, clocks developed using next-generation sequencing (NGS) data may have inherent advantages. Regional blood clocks (RegBCs), trained on RRBS data, have already demonstrated robust performance across blood and multiple tissues [98, 129, 135, 172]. Additionally, RRBS-based human blood epigenetic clocks have been developed, though further research is needed to substantiate their advantages [173, 174].

WGBS data are vast and intricately detailed, presenting considerable challenges for both interpretation and model training. In contrast to Illumina arrays, which yield an aggregate average of CpG site methylation across cells, or RRBS, which selectively targets regions with high CpG density, WGBS lacks this level of simplicity. The complexity of WGBS, combined with its high costs, has limited the development of WGBS-based clocks. Yet, the unparalleled coverage and high resolution afforded by WGBS can uncover a broader array of age-associated features, offering significant advantages in the construction of more refined clocks. Certain approaches may enable the adaptation of WGBS data for integration with existing clocks: (i) For clocks originally developed with Illumina array data, researchers could align base positions with Illumina probe sites and link these positions to probe IDs for calculation. This strategy could potentially be extended to model organism data by converting genomes to human references, although its reliability has yet to be systematically validated. (ii) For RRBS-based clocks, the process is simpler. As demonstrated by Stubbs et al., base positions and beta scores can be matched and computed using R packages, though WGBS and RRBS do not always cover identical CpG sites, which can result in missing values [129, 175].

The cost of using WGBS and RRBS to build epigenetic clocks remains prohibitively high. Developing more economical, standardized, high-throughput methods would greatly facilitate the construction of epigenetic clocks. Compared to these sequencing methods, BBA-seq has demonstrated relatively accurate age prediction, enabling the development of epigenetic clocks from a single DNA strand. This approach, though, may be better suited for targeted epigenetic studies of specific loci or detailed analysis of small sample sets. [176]. TIME-Seq, designed specifically for epigenetic clocks, offers a scalable approach for large studies, reducing costs by over 100-fold compared to traditional Illumina arrays or RRBS. Griffin et al. used TIME-Seq to develop seven mouse clocks and one human clock, which demonstrated high reliability across blood, liver, and skin tissues, with a median absolute error of 3.39 years in human blood samples [177].

While clocks constructed from large datasets inspire confidence in their robustness, the sheer volume of methylation data presents the challenge known as the “curse of dimensionality.” The Illumina 450K array spans over 450,000 sites, and the Illumina EPIC array (or 850K) nearly doubles this number. Reduced Representation Bisulfite Sequencing (RRBS) extends to millions of CpG sites, whereas Whole-Genome Bisulfite Sequencing (WGBS) encompasses almost ten times as many. Training models on such expansive datasets introduce substantial amounts of irrelevant data and noise, complicating the analytical process. Techniques for dimensionality reduction offer a way to mitigate issues like noise and overfitting. Despite the extensive evolution of epigenetic clocks, whether to prioritize comprehensive datasets (e.g., WGBS or RRBS) or more streamlined options (e.g., Illumina arrays or TIME-Seq) remains an unresolved question in clock development. We expect that methods like TIME-Seq will provide fresh insights into refining epigenetic clock construction. Equally important is the data processing strategy; by employing advanced dimensionality reduction techniques and harnessing deep learning algorithms, researchers may develop clocks that are exceptionally sensitive to specific aging signatures, potentially overcoming existing limitations and achieving greater precision [178].

## 9. The New Era of Epigenetic Clocks

CpG sites form the cornerstone of epigenetic clock construction, and the selection of these sites significantly influences the clock’s characteristics and robustness. Existing first- and second-generation clocks are predominantly constructed based on purely correlative models, targeting CpG sites with the strongest methylation association with age, regardless of whether these sites have causal relationships with aging [179-181]. Consequently, such models often include CpG sites that are mere bystanders to age-related changes rather than drivers of the aging process. This lack of causal insight limits the explanatory power of these clocks regarding the mechanisms of aging, reducing their applicability for interventions and therapies [180]. Over and beyond, correlation-based models struggle to distinguish causal relationships from confounding factors. Environmental influences, lifestyle factors, or diseases that independently affect both DNA methylation and age—but not aging itself—may be erroneously incorporated into predictions, undermining the stability and accuracy of the model under varying health or environmental conditions[182]. These limitations may partially explain the shifts observed when applying such models to datasets outside their training cohorts.

To address these challenges, Ying et al. developed a causality-enriched epigenetic clock, termed CausAge, which focuses on CpG sites with causal relationships to aging. They further refined this approach by identifying adaptive and deleterious age-related differentially methylated CpG sites. Through Mendelian randomization analysis, these causal CpG sites were classified and used to construct two distinct clocks: AdaptAge and DamAge [138]. These specialized clocks demonstrated superior predictive accuracy for aging-related phenotypes, mortality, and protective adaptations. Notably, DamAge exhibits higher robustness in capturing the influence of age-related conditions compared to current first- and second-generation clocks, emphasizing the value of separating markers of “damage” and “adaptation” in clock development [179].

Tailoring epigenetic clocks to specific objectives, such as predicting biological age, mortality risk, or aging acceleration factors, may yield more precise results. For instance, developing clocks sensitive to intervention effects or focused on evaluating specific outcomes could benefit from a streamlined selection of CpG sites. Including extraneous CpG sites not only increases the risk of overfitting but may also diminish the accuracy of predictions [178]. Another critical factor is the stochastic and spontaneous nature of DNA methylation changes over time [183, 184]. Echoing the principle of ‘horses for courses,’ the development of purpose-driven epigenetic clocks tailored to specific applications can significantly enhance their precision and reliability, particularly in specialized domains and tasks [12]. This highlights the importance of purpose-driven clock design and the potential of specialized models to improve precision [185].

Many existing clocks share overlapping CpG sites. Still, there has been no systematic analysis of the functional relevance of these shared sites. A deeper understanding of their roles and mechanisms may inform more targeted clock development. Alongside this, while non-CpG methylation sites (e.g., CHG and CHH) remain underexplored in animals, emerging research suggests intriguing possibilities. In the mouse genome, approximately 0.32% to 0.68% of CHG and CHH sites exhibit age-related methylation changes [186, 187]. Given the vast number of CHG and CHH sites across the genome, this percentage corresponds to over 2 million age-associated sites in mice. These findings suggest that non-CpG methylation may harbor valuable insights, warranting further exploration. Expanding research into non-CpG methylation sites could uncover novel mechanisms and broaden the scope of epigenetic clock development, opening up significant opportunities for future investigation [12].

## 10. Feasibility of Developing Epigenetic Clocks Using Other Data Types

Epigenetic functions as a molecular chronicle, encoding an individual’s lifetime experiences, environmental exposures, and lifestyle influences. Epigenetic clocks, grounded in theoretical models, have consistently surpassed traditional biomarkers in predicting risk and estimating lifespan, displaying remarkable accuracy across diverse applications [8, 14]. This reliability has positioned epigenetic clocks as essential tools for large-scale population studies, clinical trials, and evaluations of anti-aging interventions. In studies examining the interplay between health and aging, epigenetic clocks provide insights into both lifespan prediction and disease susceptibility, while also serving as dynamic indicators of how interventions—such as dietary adjustments and pharmacological therapies—impact biological age [70, 153, 188]. Although DNA methylation contributes significantly to this precision, it represents only a part of the aging process. A deeper understanding of other transcriptional regulators and epigenetic modifications will be essential for constructing a more holistic model that fully captures the intricacies of aging biology.

RNA methylation has emerged as an exceptionally informative epigenetic marker, significantly shaped by aging and external environmental factors. Elevated levels of RNA methylation are particularly pronounced in the central nervous system, where they exert substantial influence over pathways implicated in neurodegenerative diseases, underscoring their potential relevance in the pathophysiology of these conditions [189-191]. Acting as a critical intermediary between epigenetic modifications and post-transcriptional regulatory networks, RNA methylation orchestrates multiple intricate layers of gene expression. By modulating processes such as mRNA stability, splicing, and translation efficiency, it plays an indispensable role in fine-tuning cellular functions and developmental pathways. It governs mRNA fate and cellular differentiation while exerting a dynamic influence on RNA translation [192-194]. Compared to 5mC, the predominant marker in DNA methylation, m-A—the principal marker in RNA methylation—undergoes modifications at a markedly accelerated rate, allowing it to capture rapid and dynamic molecular changes reflective of real-time biological fluctuations. This unique characteristic not only underscores its capacity to provide a more immediate snapshot of physiological states but also reveals its stronger correlations with neurodegenerative disorders and metabolic syndromes [195, 196]. These attributes underscore the potential of RNA methylation as a foundation for constructing epigenetic clocks, though several technical and interpretative challenges must still be addressed.

**Figure 3.**
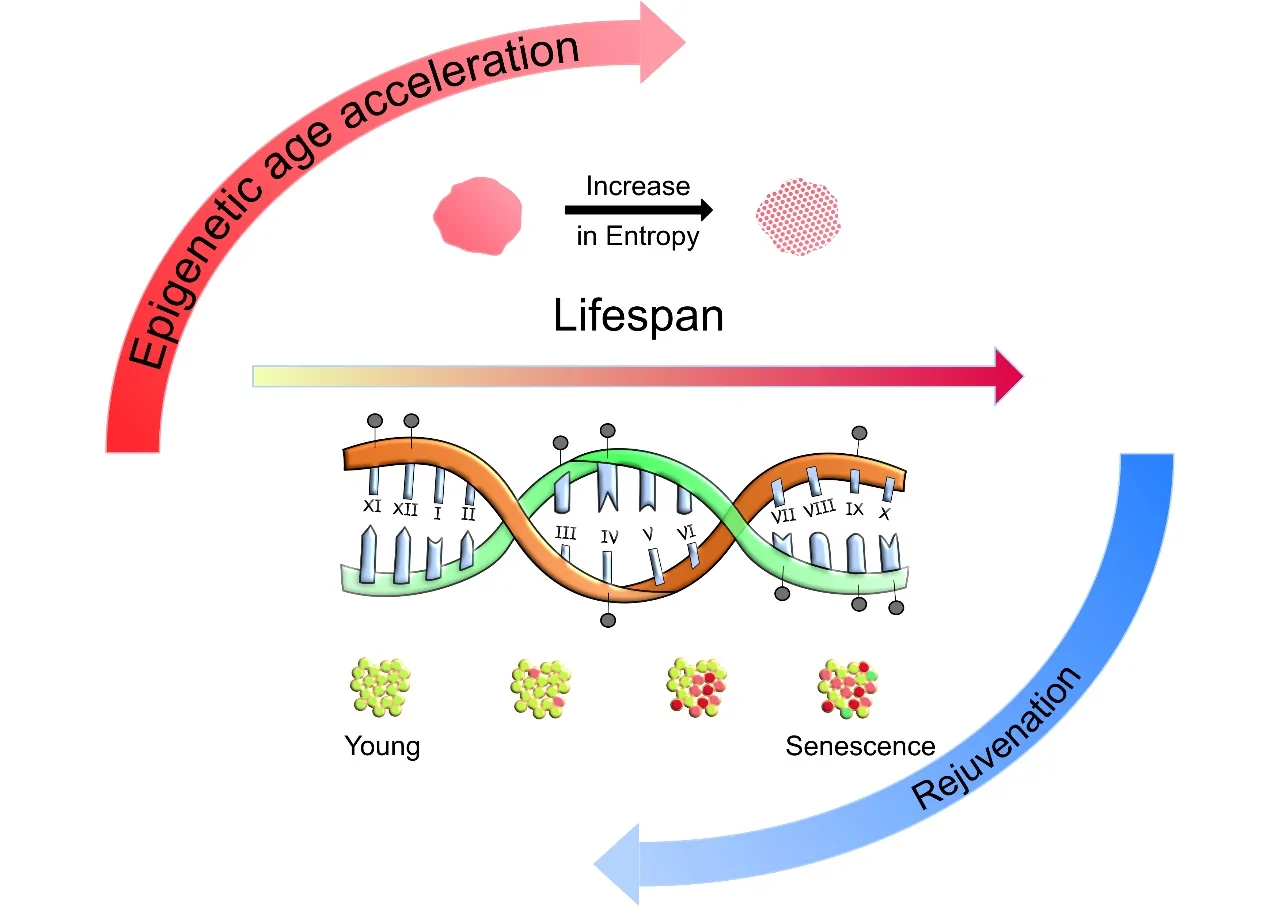
**As the lifespan progresses, DNA methylation patterns become increasingly disordered, leading to gradual cellular aging—an indicator of rising entropy**. Epigenetic markers can capture these changes, reflecting not only accelerated aging beyond chronological age but also rejuvenation effects resulting from targeted interventions. The gray pins indicate methylation sites, while red represents aging cells.

The detection of m-A remains less advanced compared to that of 5mC, complicated by its greater complexity and highly dynamic nature, with many aspects of RNA methylation mechanisms yet to be fully elucidated [197, 198]. m-A modifications are also inherently transient, persisting for only a few hours to days, and are significantly less stable than 5mC in DNA [199]. As detection technologies continue to improve in resolution and accuracy, there is hope that m-A can soon be examined at an even finer scale, offering a fresh perspective on the epigenetic landscape of aging and opening new avenues for the development of epigenetic clocks (Fig. 3).

## 11. Re-evaluating Epigenetic Clocks

The information provided by epigenetic clocks extends beyond mere markers of chronological age. While the outputs of current clocks are not inherently difficult to interpret, understanding how to correctly interpret these results is crucial for making meaningful predictions.

Beyond the absolute value of epigenetic age, “epigenetic acceleration” is often used to assess individual aging. For first-generation clocks, a predicted age that exceeds chronological age suggests accelerated aging, while a lower predicted age implies a younger biological state. Second-generation clocks, which integrate various health-related indicators and disease risk information, offer a more comprehensive assessment of health. In this context, faster epigenetic acceleration indicates a higher aging rate and potentially greater health risks, with specific risks depending on the characteristics of the training data used to build each clock. This acceleration is measured by the difference between predicted and chronological age, often referred to as the Age Acceleration Residual (AAR). From a modeling perspective, residuals represent an evaluation metric for “bias-free” prediction; as some studies have suggested, lower residuals imply a more accurate prediction [28].

In the realm of epigenetic clocks, achieving a zero AAR may be an impractical goal, given the difficulties in assembling large, low-heterogeneity datasets for clock construction and the lack of a universally accepted standard for “optimal” methylation states at each CpG site (or other markers) across all age groups [20, 130]. Striving for zero residuals may also be unnecessary. The primary function of epigenetic clocks is to quantify the divergence between biological and chronological age, making the residual a meaningful component of the clock’s utility. Similarly, correlation alone does not equate to causation, nor does it guarantee the clock’s accuracy. The true predictive strength of an epigenetic clock lies in its deviation from chronological age. If a clock were to match chronological age consistently, its value would be diminished—why calculate biological age if it offers no additional insights? The capacity of epigenetic clocks to identify outliers should be a fundamental criterion in assessing their predictive effectiveness. While traditional metrics, such as mean absolute error and correlation, provide valuable insights, an exclusive reliance on these measures’ risks offering a partial understanding. The development of an epigenetic clock with precise accuracy remains a formidable challenge, as there is no universally recognized benchmark for defining a “normal” methylation profile. Methylation patterns primarily reflect the body’s adaptive responses to changing environmental conditions [182]. Consequently, an ideal epigenetic clock would inherently show residuals even among healthy individuals, rather than aiming for a zero-residual benchmark. Such an objective is ambitious and may, in fact, pose a greater challenge than creating a “zero-residual clock.” Perhaps the day we can define methylation with the same clarity as we define normal physiological states will mark the true maturation of epigenetic clocks.
